# Reported outcomes in transsphenoidal surgery for pituitary adenomas: a systematic review

**DOI:** 10.1007/s11102-023-01303-w

**Published:** 2023-03-02

**Authors:** Hugo Layard Horsfall, Alistair Lawrence, Ashwin Venkatesh, Ryan T. S. Loh, Ronie Jayapalan, Olympia Koulouri, Rishi Sharma, Thomas Santarius, Mark Gurnell, Neil Dorward, Richard Mannion, Hani J. Marcus, Angelos G. Kolias

**Affiliations:** 1 Division of Neurosurgery, Department of Clinical Neurosciences, Addenbrooke’s Hospital and University of Cambridge, Cambridge, UK; 2 Department of Neurosurgery, The National Hospital for Neurology and Neurosurgery, Queen Square, London, W1CN 3BG UK; 3 Wellcome/EPSRC Centre for Interventional and Surgical Sciences, University College London, London, UK; 4 Department of Neurosurgery, Royal London Hospital, London, UK; 5 Clinical School of Medicine, University of Cambridge, Cambridge, UK; 6 Metabolic Research Laboratories, Institute of Metabolic Science and Department of Medicine, University of Cambridge and Addenbrooke’s Hospital, Cambridge, UK; 7 Department of Otolaryngology, Addenbrooke’s Hospital and University of Cambridge, Cambridge, UK

**Keywords:** Neurosurgery, Pituitary, Transsphenoidal, Outcome, Adenoma, Core outcome sets

## Abstract

**Purpose:**

Transsphenoidal surgery is an established treatment for pituitary adenomas. We examined outcomes and time points following transsphenoidal surgery for pituitary adenoma to identify reporting heterogeneity within the literature.

**Methods:**

A systematic review of studies that reported outcomes for transsphenoidal surgery for pituitary adenoma 1990–2021 were examined. The protocol was registered a priori and adhered to the PRISMA statement. Studies in English with > 10 patients (prospective) or > 500 patients (retrospective) were included.

**Results:**

178 studies comprising 427,659 patients were included. 91 studies reported 2 or more adenoma pathologies within the same study; 53 studies reported a single pathology. The most common adenomas reported were growth hormone-secreting (n = 106), non-functioning (n = 101), and ACTH-secreting (n = 95); 27 studies did not state a pathology. Surgical complications were the most reported outcome (n = 116, 65%). Other domains included endocrine (n = 104, 58%), extent of resection (n = 81, 46%), ophthalmic (n = 66, 37%), recurrence (n = 49, 28%), quality of life (n = 25, 19%); and nasal (n = 18, 10%). Defined follow up time points were most reported for endocrine (n = 56, 31%), extent of resection (n = 39, 22%), and recurrence (n = 28, 17%). There was heterogeneity in the follow up reported for all outcomes at different time points: discharge (n = 9), < 30 days (n = 23), < 6 months (n = 64), < 1 year (n = 23), and > 1 year (n = 69).

**Conclusion:**

Outcomes and follow up reported for transsphenoidal surgical resection of pituitary adenoma are heterogenous over the last 30 years. This study highlights the necessity to develop a robust, consensus-based, minimum, core outcome set. The next step is to develop a Delphi survey of essential outcomes, followed by a consensus meeting of interdisciplinary experts. Patient representatives should also be included. An agreed core outcome set will enable homogeneous reporting and meaningful research synthesis, ultimately improving patient care.

**Supplementary Information:**

The online version contains supplementary material available at 10.1007/s11102-023-01303-w.

## Introduction

Pituitary adenomas are common, accounting for 10–25% of intracranial neoplasms [1, 2]. From the third decade onwards, pituitary adenoma is the most common cause of an intrasellar mass [1]. Pituitary adenomas are benign tumours that are broadly categorised as functioning (hormone secreting) and non-functioning (non-secreting) and either microadenomas (< 10 mm) or macroadenomas (≥ 10 mm). Optimal management depends on numerous factors, including the clinical findings, endocrine profile, and imaging characteristics. If surgical resection is indicated, this can be performed via an endonasal transsphenoidal approach employing an endoscopic or microscopic technique.

Surgical treatment of pituitary adenoma is associated with good neurological and endocrinological recovery in the majority of patients [3]. Disease recurrence does occur and often requires subsequent management [4, 5]. As such, it is pertinent to develop an evidence-based approach for the treatment of this common condition. However, and notwithstanding the numerous studies investigating the management and outcomes of pituitary adenoma, there remain numerous barriers that prevent the development of rigorous evidence-based management strategies. Heterogeneous data collection remains a global challenge for the scientific community, contributing to significant research wastage, inefficiency and contributes to the ever escalating costs of biomedical research [6]. Heterogeneity in baseline variables and outcomes reporting stifles pooled analysis and result in imperfect meta-analyses [7].

A core outcome set (COS) is “an agreed standardized set of outcomes that should be measured and reported, as a minimum, in all clinical studies and trials in specific areas of health or health care”[8]. COS aim to reduce heterogeneity in reporting, facilitate meta-analysis, and reduce the risk of reporting bias [9, 10]. Examples of successful COS development and implementation within the literature are demonstrated in rheumatoid arthritis, stroke and traumatic brain injury [11–13]. Central to COS development is patient-public engagement, with patients suffering with a given condition being consulted during the development process to ensure the outcomes are aligned with their perspectives. In addition, funding bodies, such as the National Institute for Health Research in the UK recommend the use of COS, if available, for grant applications for clinical trials [14].

The development of a core outcome set is a multistep process. Firstly, a systematic appraisal of the existing literature identifies the range of outcome measures reported for a particular disease and quantifies the heterogeneity. The second step involves deriving the core outcome set using a structured consensus process involving all relevant stakeholders, including clinicians, academics, allied health care professionals, patients, and carers. International guidance and development standards are championed by organisations such as the Core Outcome Measures in Effectiveness Trials initiative on the development process [10, 15].

This systematic review aims to explore the range and heterogeneity of outcomes and time-points reported in studies where patients underwent transsphenoidal pituitary adenoma surgery. Our study represents the initial step in the process of developing a consensus-based COS for pituitary adenoma, with the overarching ambition to standardise outcome reporting.

## Methods

### Protocol and registration

The protocol for this systematic review was registered prospectively with OSH Registries (www.osf.io; https://doi.org/10.17605/osf.io/v9a6j). This review was conducted in accordance with the preferred reporting items for systematic reviews and meta-analyses (PRISMA) Guidelines [16]. Ethical approval was not required for this study as it was a retrospective analysis of published literature to inform future research.

### Search strategy

A search of Medline and Embase databases was performed inclusive of 1990–February 2021 to identify studies containing pituitary adenoma, an intervention and outcome. We searched all studies describing the transsphenoidal approach for pituitary adenoma (Supplementary 1).

### Eligibility criteria

Randomized controlled clinical trials, prospective cohort studies (> 10 patients), retrospective studies (> 500 patients) were included of patients with pituitary adenoma undergoing operative transsphenoidal intervention as the primary treatment strategy, in a similar fashion to other systematic reviews [17, 18]. Case reports, studies describing medical-only treatment therapies, systematic reviews and studies reporting transcranial operative approaches were excluded. Only studies written in English were included.

### Study selection

Assessment for eligibility was performed independently in duplicate by three authors (HLH, AL, RJ) in a blinded manner. Any disagreement was resolved by discussion—overseen by the senior author (AK).

### Data extraction

Data was extracted from full-text articles by the authors (RL, AV and HLH) using a piloted proforma Microsoft Excel (Microsoft Inc., Seattle, WA). Another author (HLH) verified 20% of randomly selected extracted studies to ensure internal validity. Baseline data for each study were collected and are listed below (“data items”). All outcomes were assigned to standardized outcome terms (to account for variations in wording between studies), noting the presence of any accompanying definition and timepoints captured in follow up studies.

### Data items

A pilot extraction template was utilised for the first 10 studies. The senior author reviewed the extraction template, which was iteratively developed to ensure a broad scope of outcomes were captured. The final domains were extracted: (1) study details: first author, year, journal, location of study; (2) study design: study period, type of study, number of patients; (3) surgical complications: cerebrospinal fluid leak, epistaxis, intraoperative arterial injury, resection cavity haematoma, infection, return to theatre, death; (4) extent of resection: extent of resection defined, residual tumour reported; (5) radiological recurrence: imaging modality; (6) endocrine outcomes: hypopituitarism, functioning tumour remission post-operatively, diabetes insipidus, syndrome of inappropriate antidiuretic hormone release; (7) ophthalmic outcomes: visual improvement, visual deterioration, visual acuity, other ophthalmic variable; (8) nasal outcomes; (9) quality of life outcomes including reporting scales used. We also assessed if heterogeneity in outcome reporting was found associated with different publication eras. For this, outcome reporting was compared between 1993–1998 and 2016–2021.

For each outcome domain, we established if it was reported within the study. If the outcome was reported, it was established if that was the primary outcome of the study. The length of follow up was also extracted for each outcome domain. This was recorded as time intervals: up to discharge, up to 28 days, up to 6 months, up to 1 year, and annually.

### Analysis

Descriptive statistical analysis was performed using Microsoft Excel (Microsoft Inc., Seattle, WA).

### Risk of bias assessment

As there will be no synthesis of results data in this systematic review, an assessment of the methodological quality of the included studies was outside the scope of this study and not performed.

## Results

### Study demographics

**Fig. 1:**
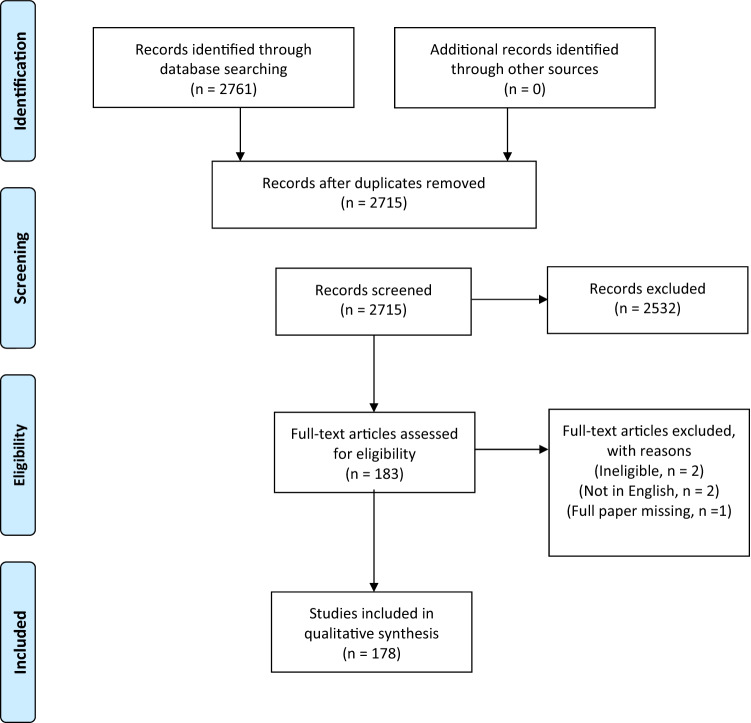
PRISMA Flow Diagram demonstrating inclusion of studies

A total of 178 studies were eligible for inclusion, comprising 427,659 patients (Fig. 1). There were 52 retrospective studies (n = 52/178, 29%), 118 prospective studies (n = 118/178, 66%) and 9 randomised controlled trials (n = 9/178, 5%) (Table 1). One study included both retrospective and prospective patients. There were 14 studies from 1990 to 1999 (n = 14/178, 8%), 35 studies from 2000 to 2009 (n = 35/178, 20%), and 129 studies from 2010 to 2021 (n = 129/178, 72%) (Table 1). Ninety-one studies reported 2 or more pathologies and 53 studies reported only one pathology. Twenty-seven studies did not specify the pituitary adenoma pathology. Non-functioning pituitary adenomas were reported in 101 studies. Functioning pituitary adenomas reported were growth hormone secreting (106 studies), ACTH-secreting (95 studies), prolactin-secreting (80 studies) and TSH-secreting (31 studies). Transcription factors were rarely reported. From 2015 onwards, only 1 paper out of a possible 98 reported transcription factors.

**Table 1:** Breakdown of studies which met the inclusion criteria by decade, number of patients and study type

Decade & study type	1990–1999			2000–2009			2010–2021			Total
No. of patients	R	P	RCT	R	P	RCT	R	P	RCT	
1–250	0	9	2	0	19	3	0	73	4	110
251–500	0	0	0	0	1	0	0	6	0	7
501–1000	2	0	0	5	2	0	18	6	0	33
> 1000	1	0	0	5	1	0	21	1	0	29
Total	3	9	2	10	23	3	39	86	4	179

Note a 2004 study with > 1000 patients included both retrospective and prospective data, and has been represented as two separate studies in this table

*R* retrospective cohort study, *P* prospective cohort study, *RCT* randomised controlled trial

### Outcome domains and length of follow up

**Fig. 2:**
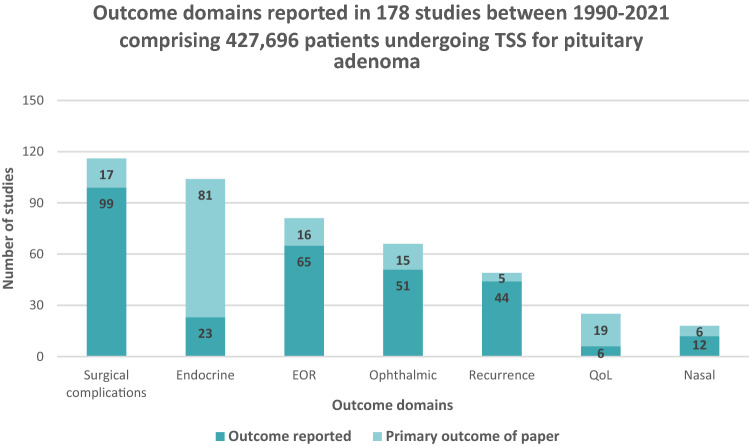
Outcome domains reported by studies, and if outcome domain reported was the primary outcome of the study. TSS transsphenoidal surgery, EOR extent of resection, QoL quality of life

Surgical complications were reported in 116 studies (65%) (Table 2, Fig. 2), and was the most frequently reported outcome. Endocrine outcomes were reported in 104 studies (58%), followed by extent of resection (81 studies, 46%), ophthalmic (66 studies, 37%), recurrence (49 studies, 28%), quality of life (25 studies, 14%), and nasal outcomes (18 studies, 10%) (Table 2). The outcome domain associated with the most frequently defined follow up length was endocrine outcomes (56 studies, 54%) and extent of resection (39 studies, 48%) (Table 2). The most frequently defined follow up length across all outcome domains for follow up was up to 6 months (64 studies) and over 1 year (69 studies). Quality of life and nasal outcomes had the highest percentage of total studies reporting the outcome theme and time point for follow up, 60% and 61% respectively, whilst only 15% of surgical complications reported had a defined follow up (Table 2).

**Table 2:** Summary of outcome domains reported and length of follow up

Outcome domain	Total # reporting outcome	Total # reporting length of follow up	Specific length of follow up reported				
			Discharge	Up to 30 days	Up to 6 months	Up to 1 year	Over 1 year
Surgical complications	116	17	3	8	3	0	3
Endocrine	104	56	4	8	19	4	21
EOR	81	39	0	4	17	4	14
Ophthalmic	66	22	0	2	10	5	5
Recurrence	49	28	0	1	4	1	22
Quality of life	25	15	2	0	7	5	1
Nasal	18	11	0	0	4	4	3
Total			9	23	64	23	69

Total number of outcome domains and follow up reported is greater than total number of studies, as each study could report numerous outcome domains

*EOR* extent of resection

### Surgical complications

**Fig. 3:**
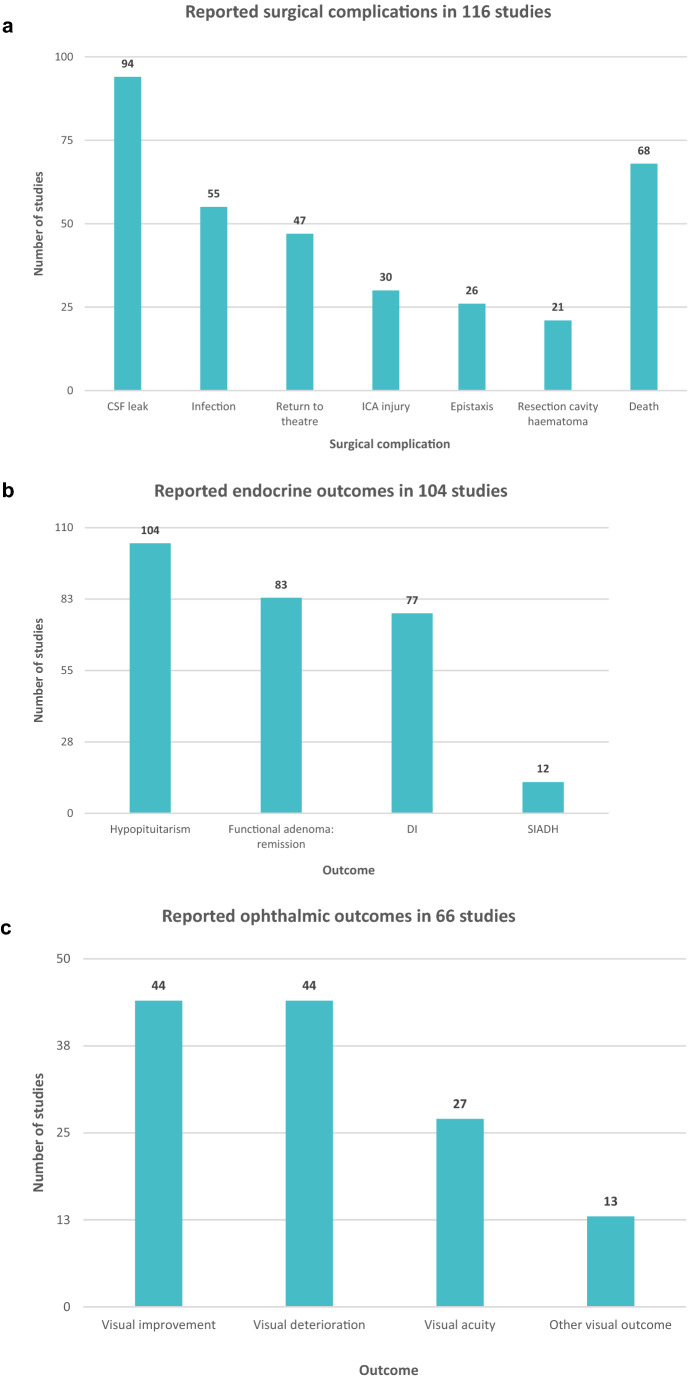
Summary of surgical complications, endocrine and visual outcomes. **a** Summary of surgical complications outcomes, **b** Summary of endocrine outcome, **c** Summary of visual outcomes

Surgical complications were reported in 116 studies (65%) and was the primary outcome in 17 studies (10%) (Table 2; Fig. 2). Post-operative cerebrospinal fluid (CSF) leak was the most reported surgical complication (n = 94/116, 81%) (Fig. 3). Most studies reporting CSF leak lacked any definition—only five studies defined post-operative CSF leak. 40 studies reported that the CSF leak required intervention, of which 26 reported the mechanism of intervention. Death was the next most reported surgical complication (n = 68/116, 57%). Other reported surgical complications included infection (including meningitis) (n = 55/116, 47%), return to theatre (n = 47/116, 41%), length of hospital stay (n = 41/116, 35%), intraoperative arterial injury (n = 30/116, 27%), epistaxis requiring intervention (n = 26/116, 22%), and resection cavity haematoma (n = 21/116, 18%) (Fig. 3a).

### Endocrine outcomes

Endocrine outcomes were reported in 104 studies (n = 104/178, 58%), and were the primary outcome in 23 studies (n = 23/178, 13%) (Table 2, Fig. 2). Post-operative hypopituitarism was reported in every study that discussed endocrine outcomes (104/104, 100%). Hypopituitarism included new hormonal deficits, and patients requiring human replacement post-operatively. For functioning adenomas, 83 studies reported remission from surgery (n = 83/104, 80%). AVP deficiency (cranial diabetes insipidus (DI)) as a post-operative complication was reported in 77 studies (n = 77/104, 74%). Of the 77 studies that reported DI, only 7 studies explicitly defined DI. Twelve studies reported syndrome of inappropriate antidiuretic hormone (SIADH) secretion (n = 12/104, 12%), with only one study defining SIADH (Figs. 2, 3b).

### Ophthalmic outcomes

Ophthalmic outcomes were reported in 66 studies (n = 66/178, 37%), and were the primary outcome of 15 studies (n = 15/178, 8%). Both visual improvement and visual deterioration were reported in 44 studies (n = 44/66, 67%) (Fig. 3c). The VFQ-25 was used in 2 studies. Visual fields were reported in 41 studies (n = 41/66, 62%). Of the studies reporting visual fields, 19 studies (n = 19/41, 46%) reported the assessment measure used (Humphrey, 11; Goldmann, 2; other, 3; did not specify, 3). Visual acuity was reported in 27 studies (n = 27/66, 41%). Eight studies reported the assessment measure (Snellen chart, n = 8/8, 100%). Other ophthalmic outcomes were reported in 13 studies and included diplopia, colour vision, optic disc grading and retinal nerve fibre layer thickness.

### Extent of resection

The extent of resection (EOR) following transsphenoidal surgery was reported in 81 studies (n = 81/178, 46%), and was the primary outcome in 16 studies (n = 16/178, 9%) (Table 2, Fig. 2). Only 10 studies defined EOR, with heterogenous definitions such as “*dichotomized into GTR and STR, formal volumetric analysis to determine the percentage of tumour removed and the volume of residual tumour*”, or “*percentagewise reduction of residual volume to baseline tumour volume on pre-operative MRI*”. Gross total resection (GTR) was also only defined in 10 studies. Subtotal resection (STR) was defined in 9 studies. There were six different definitions of STR reported related to residual tumour (> 80% removed, 50–80% removed, > 15% residual lesion, < 90% resected, < 90% of the tumour removed) or radiological findings (residual tumour had to be detected on at least two consecutive thin-cut MRI slices in one imaging plane and confirmed on a slice in another imaging plane).

### Radiological recurrence

Radiological recurrence was reported in 49 studies (n = 49/178, 28%), and was the primary outcome in 5 studies (n = 5/178, 3%). Imaging modality of recurrence was reported in 28 studies (n = 28/49, 56%), of which 27 studies reported utilising magnetic resonance imaging (MRI) and one study reported MRI and computed tomography imaging. Only five studies defined recurrence explicitly, which all related to new tumour growth on MRI.

### Nasal outcomes

Nasal outcomes describe the sinonasal symptoms and signs related to the transsphenoidal approach, such as nasal congestion or discharge. Nasal outcomes were reported in 18 studies (n = 18/178, 10%), and were the primary outcome of 6 studies (n = 6/178, 3%). Nine studies reported specific validated nasal outcome scoring systems: SNOT 22 (5 studies); Sniffin’ Sticks (3 studies); UPSIT (2 studies); BAST 24 (1 study); NSS (1 study); and SIT 40 (1 study).

### Quality of life and psychological outcomes

Quality of life (QoL) and psychological outcomes were reported in 25 studies (n = 25/178, 14%), and were the primary outcome of 19 studies (n = 19/178, 11%). In total, 34 different questionnaires were used. Seven questionnaires were used more than once: SF36 (9 studies); EQ5D (4 studies); Self-rating Anxiety Score (3 studies); Self-Rating Depression Score (3 studies); ASBQ (3 studies); AcroQoL (2 studies); and EORTC-QLQ-E30 (2 studies). In the years 2000–2009, 6 of 44 studies (14%) were published that reported QoL outcomes, compared to the years 2010–2020 where 19 of 133 studies (14%) reported QoL outcomes. Fifteen studies reported healthcare costs associated with pituitary adenoma resection.

### Heterogeneity in outcome reporting across two eras (1993–1998 & 2016–2021)

Comparing 1993–1998 with 2016–2021, surgical complications were reported in 62% versus 61% respectively, endocrine outcomes 23% versus 51% respectively, extent of resection 23% versus 38% respectively, ophthalmic outcomes 31% versus 35% respectively, recurrence 38% versus 15% respectively, quality of life 8% versus 24% respectively, and nasal outcomes 0% versus 13% respectively.

## Discussion

### Principal findings

Here, we present the first study evaluating the outcomes reported for patients undergoing transsphenoidal surgery for pituitary adenoma. This study included 178 studies over a 30 year period and included almost 430,000 patients. We aimed to assess general outcome domains to get a flavour of the literature, as opposed to specific outcome measures and meta-analysis. Our data demonstrates significant heterogeneity in the reporting of outcomes, and length of follow up across all domains. This data provides rationale for the international community delivering pituitary specialist services to collaborate and a consensus-based approach on the most important outcomes to facilitate improved patient care.

### Outcome heterogeneity in transsphenoidal pituitary adenoma surgery

Surgical complications were the most frequently reported outcome domain in patients undergoing transsphenoidal surgery for the pituitary adenoma in the literature over the last 30 years (Fig. 3; Table 2). This complication is relevant to both functioning and non-functioning pituitary adenomas. Post-operative CSF leak is a well-recognised complication, and accordingly was reported in 94 studies. However, only 5 of the 94 studies provided a definition. Methods to manage the post-operative CSF leak is known to be heterogenous, surgeon and unit specific, but a recent UK-wide study utilised a standardised definition of CSF leak to positive effect.[19]. Other frequently reported surgical outcomes included infection (55 studies), return to theatre (47 studies), internal carotid artery injury (30 studies) and epistaxis (26 studies). It is appreciated that differing surgical outcomes have different significance in the overall patient pathway including length of stay, and longer-term mortality and morbidity. However, having an agreed minimum of reported outcomes would provide clinical utility.

Endocrine outcomes were the second most reported outcomes, in 104 studies. Hypopituitarism, which included new hormonal deficits, and patients requiring human replacement post-operatively, was reported in all studies reporting endocrine outcomes (100%). Remission and DI rates were described in the majority of studies reporting endocrine outcomes (80% and 74%, respectively). Less well reported was SIADH (12%) (Fig. 3b). Endocrine disequilibrium is of paramount importance. In many cases, post-operative endocrine deficits are permanent and therefore associated with a lifelong requirement to take daily hormone replacement such as growth hormone or testosterone. Hypopituitarism is known to be associated with reduced quality of life, increased morbidity and cardiovascular mortality [20, 21]. As such, both patients and clinicians need to be aware of the risk of this potential complication following transsphenoidal surgery. Further, a thorough evaluation is required, interpreted in the correct clinical context and including dynamic endocrine testing where indicated. Otherwise, it may be unclear if post-operative deficits are acute. The complexity of post-operative endocrine management is hindered by a lack of standardised endocrine assessment both pre- and postoperatively.

Ophthalmic outcomes are essential for patients with pituitary adenomas [22]. 66 studies reported ophthalmic outcomes, of which 44 studies reported subjective visual improvement or deterioration. Ophthalmic outcomes were objectively analysed in some of the papers: 41 studies reported visual fields and 27 studies reported visual acuity. Of the studies reporting visual fields, this was assessed using Humphrey (11 studies) and Goldmann: (2 studies). Visual acuity was reported in 27 studies, but only 8 studies specified Snellen chart results. The discrepancy and heterogeneity of ophthalmic outcome reporting in the literature is demonstrated by our data. Patient-reported outcome measures are increasingly used to access pain, disability, physical function, and mental status to quantify effectiveness of surgical intervention in other fields of neurosurgery (e.g. cervical myelopathy) [23]. The 25-item National Eye Institute Visual Function Questionnaire (VFQ-25) is a validated, comprehensive assessment tool to demonstrate vision-related quality of life [24]. However, only 2 studies utilised the VFQ-25. Using ophthalmic outcomes as an example, our study’s findings provide tangible data that reporting of outcomes following pituitary adenoma surgery can utilise existing patient-reported metrics and that authors should provide objective assessments of outcomes. This call-to-arms would be centralised through a consensus-based core outcome set, where the outcome and tool to measure the outcome are decided by expert, patient, and stakeholder consensus.

Significant changes in pituitary tumour management have occurred over the time period of studies included in this work. Subgroup analysis of opposing eras highlights that the absolute numbers of studies and the frequency with which they report pituitary surgery outcomes have increased unanimously across domains with time (Table 3). This suggests that whilst domain heterogeneity appears to be improving over time, consensus outcome reporting remains a critical objective for future studies in this field.

**Table 3:** Study outcome reporting (absolute number and % of total studies) in different domains across two different eras (1993–1998 & 2016–2021)

	1993–1998 (13 studies, 3187 patients)		2016–2021 (93 studies, 278,272 patients)	
Outcome domain	Studies reported	% Studies reported	Studies reported	% Studies reported
Complications	8	62	57	61
Endocrine	3	23	47	51
EOR	3	23	35	38
Ophthalmic	4	31	33	35
Recurrence	5	38	14	15
Quality of life	1	8	22	24
Nasal	0	0	12	13

*EOR* extent of resection

### Length of follow up

Length of follow up is critical for all clinical studies, and particularly important for evaluating outcomes following surgery. For many outcomes identified, a short follow up is not meaningful. For example, one series demonstrated that Cushing’s disease patients undergoing TSS had a mean time to relapse of 5.3 years, with an average length of follow up of 9.6 years [25]. However, this review established that of the 104 studies that reported endocrine outcomes, only 56 studies reported time points (Table 2). Of these studies, only 21 studies reported follow up of over 1 year. Recurrence had improved reporting within the literature—although it was less frequently reported in all studies (49 studies), 22 studies reported follow up time points of > 1 year. Similarly, improvement in visual fields can occur up to 5 years following treatment [26]. Ophthalmic outcomes (Figs. 2, 3c) were reported in 66 studies, however only 22 studies specified length of follow up and 12 of these studies the maximum length of follow up reported was 1 year. Conversely, surgical complications were the most reported outcome (116 studies) but had the least number of studies that reported the follow up (17 studies), of which most were < 6 months (Table 2; Fig. 2). Long-term follow up is resource-intensive, subject to numerous biases, and it is acknowledged that pituitary patients might be transferred to other services, such as local endocrine teams outside of the specialist neurosurgical centre. As such, patient *data* might be “lost” to follow-up, rather than the *patient* being lost to follow up. This highlights a common shortfall in overall research infrastructure, not just applicable to pituitary subspecialities, Further, the heterogeneity of follow up reinforces the evidence that specialist centres delivering pituitary services is not standardised.

### Core outcome sets

The selection of appropriate outcomes in clinical research requires greater attention from the scientific community if study findings are to be reliable and relevant to patients, healthcare professionals and other stakeholders making decisions regarding healthcare provision [27]. A 2014 Lancet Series ‘Research: Increasing Value, Rescuing Waste’ estimated 85% of biomedical research offering actual or potential clinical benefit was prevented due to research inefficiency [6], equating to $200 million in 2010. One of the reasons postulated to contribute to this heterogenous data collection and reporting. This is further demonstrated by Tovey in a Cochrane Review, stating problems due to inconsistencies in the outcomes reported in trials [28]. There is a wide range of outcome domains that are relevant for any given condition. A pertinent challenge is to determine which are the most important to capture. It is also important to consider how we define “outcomes”. A recent study by Young et al. [29] identified inconsistencies in how authors define, extract, group, and count trial outcomes. This potentially introduces systematic bias and contributes to research inefficiency. One method to counteract reporting bias is through international, consensus-based definition of a minimum set of outcomes that should be reported by studies in a field. This begins with a systematic review of the literature, such as the present study, followed by Delphi surveys and a final consensus meeting [9]. Systematic COS development and implementation will hopefully lead to higher-quality studies, and facilitate comparison of results to ultimately reduce research inefficiencies [30]. Importantly, international societies relating to pituitary disorders have demonstrated consensus-based strategies to define the operative phases and steps of transsphenoidal operations, demonstrating precedence and appetite within the literature [31].

### Limitations

The present systematic review has focused on the transsphenoidal approach to pituitary adenoma. Many of the included studies have reported multiple pathologies within their studies, often without separating functioning versus non-functioning pituitary adenoma. This induces reporting bias as there is heterogeneity in the outcomes for functioning and non-functioning pituitary adenoma. However, in a pragmatic, constraint-based approach, the overall result of this review still demonstrates heterogeneous reporting evident in all studies reporting transsphenoidal pituitary adenoma resection. Additionally, our aim was to assess general outcome domains to get a flavour of the literature, as opposed to specific outcome measures. Our inclusion criteria of > 500 patients in retrospective studies meant that, generally, in larger studies there is less granular data, and we could have missed small series with longer length of follow up. Having said that, our aim to address outcome domains rather than specific scales per se was achieved and our study supports the call for an outcome set, which will be derived through a consensus-based process.

## Conclusion

The present study demonstrates heterogeneity in outcome and follow up reporting for patients with pituitary adenoma undergoing transsphenoidal resection. The review represents the initial step in the development of a core outcome set which requires Delphi consensus. This would aid meta-analysis, improve the quality and consistency of reporting, reduce research wastage, and ultimately benefit patients through improved clinical services.

## Supplementary Information

Below is the link to the electronic supplementary material.

Supplementary file1 (DOCX 11 kb)

## Data Availability

The datasets generated during and/or analysed during the current study are available from the corresponding author on reasonable request.
