# Supplementary material for: Reported outcomes in transsphenoidal surgery for pituitary adenomas: a systematic review
Source: Pituitary. 2023 Mar 2;26(2):171–81. doi: 10.1007/s11102-023-01303-w (PMC10247847; doi:10.1007/s11102-023-01303-w)
Supplement: Supplementary file 1 — Supplementary file1 (DOCX 11 kb) [file 11102_2023_1303_MOESM1_ESM.docx]

**Supplementary 1**

((((pituitar*[Text Word]) AND ((tumo*[Text Word]) OR adenoma*[Text Word])) AND (((((((surger*[Title/Abstract]) OR resect*[Title/Abstract]) OR remov*[Title/Abstract]) OR excis*[Title/Abstract]) OR debulk*[Title/Abstract]) OR operat*[Title/Abstract]) OR interven*[Title/Abstract])) AND ((outcome*[Text Word]) OR endpoint*[Text Word])) NOT "case reports"[Publication Type]
